# Growth performance, histological and physiological responses of heat-stressed broilers in response to short periods of incubation during egg storage and thermal conditioning

**DOI:** 10.1038/s41598-023-50295-x

**Published:** 2024-01-02

**Authors:** Sayed A. Abdel-Fattah, Mahmoud Madkour, Mona A. Hemida, Mohamed Shourrap

**Affiliations:** 1 Poultry Production Department, Faculty of Agriculture, Ain Shams University, Shoubra El-Kheima, 11241 Cairo Egypt; 2 Animal Production Department, National Research Centre, Dokki, 12622 Giza Egypt

**Keywords:** Animal physiology, Zoology, Physiology

## Abstract

The short periods of incubation during egg storage (SPIDES) method enhances the quality of chicks and improves hatching rates. Additionally, embryonic thermal conditioning (TC) is a technique used to enhance thermotolerance in birds. Previous studies have evaluated the effects of SPIDES and embryonic TC separately. Yet, our hypothesis postulated that a synergistic effect could be achieved by integrating TC and SPIDES, thereby enhancing the broilers' resilience to thermal stress. We conducted an experiment involving 800 Ross broiler eggs, divided into two groups. The first group, referred to as S0, was maintained under standard storage room conditions and acted as our control group. The second group, known as S1, underwent a process called SPIDES for 5 h at a temperature of 37.8 ± 0.1 °C, on three occasions: days 5, 10, and 15 following egg collection. Upon reaching the 14th day of incubation (DOI), each of these primary groups was randomly subdivided into two equal subgroups. The control subgroup, designated as TC0, remained in the usual incubation conditions. Meanwhile, the other subgroup, TC1, was subjected to prenatal heat conditioning at a temperature of 39.5 ± 0.1 °C for 6 h per day, commencing on the 14th embryonic day (E) and extending until the 18th embryonic day (E). This experimental setup resulted in four distinct experimental subgroups: S0TC0, S1TC0, S0TC1, and S1TC1. The findings indicated that the combined application of SPIDES and TC had a significant positive effect on chick performance after hatching. Specifically, the (S1TC1) group exhibited the heaviest live body weight (LBW) and body weight gain (BWG) at the marketing age in comparison to the other groups. Furthermore, both SPIDES and TC had a positive influence on the relative weights of breast muscles and their histological measurements. The (S1TC1) group displayed significantly higher values in terms of the relative weight of breast muscles and the number of myocytes. In conclusion, SPIDES and TC have beneficial effects on pre- and post-hatch characteristics of broiler chicks up until the marketing age. Additionally, TC techniques improve chick performance, particularly under conditions of heat stress, and enhance the yield of breast muscle in later stages of life.

## Introduction

Storing eggs is a widely adopted procedure in farm and hatchery operations around the globe^1^. However, it has been found that extended periods of egg storage, even under optimal conditions, have negative consequences for egg quality, ultimately impacting the quality and performance of the resulting chicks^2–4^.

A natural method known as SPIDES has been derived from the behavior of chickens, where eggs that have been laid for some time are warmed up for one or more hours and then cooled down for 24 h until the next egg is laid. This process is repeated daily until the entire clutch is complete, and all eggs are incubated together starting on the same day for synchronized hatching^5^. Recently, the application of SPIDES has been shown to modify the conventional technique used in hatcheries^6,7^. This modification aims to improve egg quality following extended periods of storage, leading to enhanced chick quality and improved performance in the future^8^.

Climate change presents a significant global challenge, disrupting weather patterns and posing a threat to poultry production. Consequently, thermal stress has become a crucial limiting factor, directly affecting the well-being of birds^9^. Particularly, the broiler industry faces escalating challenges caused by heat stress (HS) due to the rising temperatures associated with global climate change^10^. To counteract the adverse effects of heat challenges, extensive efforts have been devoted to devising various approaches^11,12^. The effects of rising summer temperatures on poultry production are becoming increasingly evident as global warming escalates. Thus, it becomes essential to explore methods that can alleviate heat stress in chickens^13^. One such approach involves implementing thermal conditioning during the neonatal period of chickens, which has shown promise in enhancing their ability to tolerate high temperatures and reducing the subsequent rise in body temperature when exposed to elevated surrounding temperatures during their later stages of life^14,15^

Earlier research has primarily concentrated on studying the separate impacts of SPIDES or embryonic TC (TC). Surprisingly, no prior investigations have explored the joint influence of TC and SPIDES. Based on this gap, we developed a theory that the combination of TC and SPIDES could potentially improve broilers' resilience to heat stress. To test our hypothesis, we conducted a comprehensive assessment of productive performance, certain physiological responses, as well as histological and cytological observations of skeletal muscles in broiler chickens exposed to heat stress challenges.

## Material and methods

### Ethical statement

The authors confirm that the ethical policies of the journal, as noted on the journal's author guidelines page, have been adhered to and The experimental design and all the research protocols were approved by the Experimental Animal Care and Research Ethics Committee of Ain Shams University, Agriculture Sector Committee with ethical approval code (5-2023-8). The study was conducted following ARRIVE guidelines.

### Experimental design

A total of eight hundred Ross breeder eggs, aged 29 weeks, averaging a weight of 63.04 ± 0.58 g, were divided into two groups of equal size based on the storage time. The first group, S0 (control), was kept under specific conditions at a temperature of 15.8°C ± 0.4 and relative humidity (RH) of 75% for a period of up to 20 days. The second group, labeled as S1, experienced the SPIDES method on days 5, 10, and 15 following egg collection, as described by^8,16^. After the 20-day storage period, all eggs were transferred to an automatic setter provided by a local manufacturer, where they were subjected to an incubation protocol consisting of 37.6 ± 0.02 °C and 60.5% of RH. On the 14th day of the incubation process, we randomly divided both groups (S0 and S1) into two equal subgroups. The control subgroup (TC0) was kept under optimal conditions, while the other subgroup (TC1) was exposed to prenatal thermal conditioning at a temperature of 39.5 ± 0.1 °C and RH of 72%. This thermal conditioning was maintained consistently for 6 h per day, from 10:00 a.m. to 4:00 p.m., over a period of five consecutive embryonic days (E14–E18). As a result, four experimental sub-groups were formed, namely S0TC0, S1TC0, S0TC1, and S1TC1 as reported by^8^.

Twenty eggs were chosen at random from each of the two main groups for the purpose of measuring egg quality on the 20th day after egg collection. We measured the egg weight with a highly precise electronic balance, which had an accuracy of 0.01g. To determine the egg's shape index (SI), we applied the formulas presented by^17,18^, which involve dividing the egg's width by its length and then multiplying the result by 100.

To measure the yolk weight, The yolk was separated from the albumen and quantified using electronic scales. The yolk weight percentage was calculated by dividing the yolk weight by the egg weight and then multiplying the outcome by 100.

The yolk index (YI), invented by^19^, was computed using the measurements of yolk height and yolk diameter with the formula:

$$ {\text{YI }} = \, \left( {{\text{yolk height }}/{\text{ yolk diameter}}} \right) \, \times { 1}00. $$

We calculated the albumen weight by subtracting the combined weight of the yolk and shell from the total egg weight. Subsequently, the albumen weight percentage was obtained by dividing the albumen weight by the egg weight and multiplying the quotient by 100.

The albumin index was computed as the division of the albumin height by the average albumin width.

The pH of the albumen was promptly assessed using a pH meter that had been calibrated (H12212 pH Meter, HANNA Instruments).

The albumen height was converted into Haugh units (HU) to assess the freshness of the eggs, following the formula established by^20^ and modified by^21^

Egg weight loss percentage was calculated during the storage period (from day 3 to day 7, day 7 to day 15, and day 15 to day 20) and during the incubation period, including the first and second weeks of incubation, as well as during the thermal conditioning period (from embryonic day 14 to 18). This was determined by subtracting the initial egg weight from the final egg weight and dividing the difference by the initial egg weight. The percentage value was obtained by multiplying the result by 100, as described by^22^.

Upon hatching, the chicks were individually weighed and then distributed into four treatment groups. Each treatment group consisted of six replicate pens, with 25 birds housed in each pen.

The floor pens had dimensions of approximately 1.22 m × 2.44 m and were equipped with fresh pine shavings as the litter material. and their live body weights (LBW) were measured weekly till five weeks of age. An electronic balance with a precision of 0.1 g was used for accurate measurements. The average body weight gain (BWG), feed consumption (FC), and feed conversion ratio (FCR) were calculated, FCR was calculated as per gram feed consumed per gram weight produced (g feed/g weight gain).. The environmental temperature, relative humidity, and THI throughout the entire experimental duration as reported in the first part of this study in^8^. THI in the present study ranged from 29.5 to 33.5. This suggests that the broilers were raised in conditions characterized by significant heat stress. The weekly calculated values of THI have been mentioned^8^ (it will useful to show data in a table to see this conditions of stress after 24 days of raising, period when 33.5 THI should be stressing to the chicken broilers).

### Slaughter traits

When the chicks reached 35 days of age, we randomly chose six chicks from each of the four sub-groups. These chicks were weighed and then slaughtered using cervical dislocation. During the autopsy, the abdomen was opened, and the liver and heart were removed and weighed. The weights were recorded in grams, rounded to the nearest 0.001 g. The carcasses without internal organs were weighed individually, and the percentage of dressed carcass yield was recorded. The different breast muscles, namely the pectoralis major and pectoralis minor, were dissected and weighed. All recorded weights were proportional to the live body weight at the corresponding age.

### Blood parameters

In total, 24 blood samples were gathered, with six samples taken from each of the four sub-groups. These samples were collected to determine various plasma parameters. The concentrations of total proteins (g/dl) and albumin (g/dl) were assessed utilizing commercial kits acquired from Spectrum Diagnostics. The globulin levels were derived by deducting the plasma albumin content from the overall plasma protein concentration. Cholesterol (mg/dl), high-density lipoprotein (mg/dl), and triglycerides (mg/dl) were determined using commercial kits from Spectrum Diagnostics. The enzymatic activities of AST and ALT (U/L) were quantified through calorimetric assays using commercial kits obtained from Spectrum Diagnostics.

### Plasma hormonal assay

Triiodothyronine (T3) level was assessed using the Eliza technique and commercial Eliza kits obtained from Precheck Bio. Company, following the methodology described by^23^

### Histology and cytology of skeletal muscles

Samples representative of the left major pectoralis muscle in chicks were carefully dissected during the slaughtering process, with dimensions measuring 0.5 × 0.5 cm. The provided samples were promptly preserved by immersing them in an appropriate amount of a 10% formalin solution. To prepare these samples for examination, we followed the paraffin technique outlined in a study by^24^. Subsequently, we prepared thin sections, each measuring 4–5 microns in thickness, and affixed two sections from each sample onto glass slides. We then proceeded to stain these sections using the standard hematoxylin and eosin (H & E) staining procedure. These histological procedures were carried out at the Pathology Laboratory situated within Cairo University's National Cancer Institute in Egypt.

We examined the histological features of the pectoral muscle tissues by utilizing a low-magnification light microscope (Optica) at  4× magnification. To enhance the clarity of our findings, we captured images of specific samples using a digital camera (Samsung ES75). For precise quantification of various histological components within the pectoral muscles, we conducted histomorphometric measurements employing specialized image analysis software, OPTIKA PROVIEW © 2003–2020.

In each of the three pectoral muscle sections, we quantified the myocyte count within nine distinct fields (three fields per section) for all treatments, employing a magnification level of  10×. Additionally, we measured both the large (LD) and small (SD) diameters of 100 myocytes present on each slide for each treatment. These measurements allowed us to calculate cytometric and histometric indices, following the methodology outlined in^25^, using the formula provided.

$$ {\text{Mean thickness}} \left( {D\overline{x}\left( {\mu m} \right)} \right) = LD + SD/2 $$

$$ {\text{Shape index ratio}} \left( {SI} \right) = LD/SD $$

$$ {\text{Cross section area}} \left( {SA\left( {\mu m^{2} } \right)} \right) = LD \times SD \times \pi /4 $$

where: LD is the Myocytes' large diameter; SD is the Myocytes' small diameter; $\pi$ = 3.1416.

### Statistical analysis

The data were divided into two distinct experimental periods, each characterized by a unique experimental design. The initial experimental period extended from the storage phase up to the TC procedure. During this phase, the data were subjected to one-way analysis of variance, treating SPIDES as a fixed effect. This analysis was conducted using SAS's GLM procedure, as outlined in the following model:

$$ {\text{Y}}_{{{\text{ij}}}} = \, \mu + {\text{S}}_{{\text{i}}} + \varepsilon_{ij} $$

where: Y_ij_ is a trait of interest; *µ* is an effect of the overall mean; *Si* is a fixed effect of ith SPIDES period; $\varepsilon_{ij}$ is a random experimental error assumed NID (0, σe^2^).

During the second experimental period, which encompassed the duration from E14 until the conclusion of the study, the data underwent a two-way analysis of variance. This analysis involved treating SPIDES and TC as fixed factors and considering their interactions. The analysis was performed using SAS’s GLM procedure, and the model used is described as follows:

$$ {\text{Y}}_{{{\text{ijk}}}} = \, \mu + {\text{S}}_{{\text{i}}} + {\text{ T}}_{{\text{j}}} + {\text{ S}}_{{\text{i}}} {\text{T}}_{{\text{j}}} + \varepsilon_{{ij{\text{k}}}} $$

where: *Y*_*ijk*_ is trait of interest; *µ* is an effect of overall mean; *S*_*i*_ is a fixed effect of *i*th SPIDES period; *T*_*i*_ is a fixed effect of *j*th thermal conditioning; *S*_*|*_*T*_*j*_ is the interaction effect between S_i_ and T_j_. $\varepsilon_{ijk}$ is a random experimental error assumed NID (0, σe^2^).

To differentiate between means when applicable, we applied Duncan's multiple range test, as introduced by Duncan in 1955. For the analysis, we divided the percentages of slaughter traits by 100 and subjected them to an arcsin transformation of the square root. However, the actual percentage means are presented for clarity. In terms of statistical significance, we considered a probability level of 0.05 (p ≤ 0.05) as the threshold for acceptance.

To differentiate between means when applicable, we applied Duncan's multiple range test, as introduced by^26^. For the analysis, we divided the percentages of slaughter traits by 100 and subjected them to an arcsin transformation of the square root. However, the actual percentage means are presented for clarity. In terms of statistical significance, we considered a probability level of 0.05 (p ≤ 0.05) as the threshold for acceptance.

## Results

### Egg quality

Table 1 shows the data of the influence of SPIDES during a prolonged storage period of 20 days on measurements of both external and internal egg quality.

**Table 1:** Effect of SPIDES during the long storage period (20 days) on external and internal egg quality measurements.

SPIDES (S)		Significance
S_0_	S_1_	
Egg wt at 20 DOS (g)		
60.51 ± 0.12	62.75 ± 0.37	NS
Egg shape index (%)		
78.73 ± 1.26	79.31 ± 1.20	NS
Shell weight (%)		
8.55 ± 0.05	9.20 ± 0.24	NS
Egg surface area (mm)		
71.92 ± 0.57	73.82 ± 0.30	NS
Yolk weight (%)		
34.00^a^ ± 0.50	30.33^b^ ± 0.67	**
Yolk index (%)		
34.33^b^ ± 0.02	38.95^a^ ± 0.95	**
Albumen weight (%)		
57.37^b^ ± 0.27	60.12^a^ ± 0.27	**
Albumen index (%)		
5.80^b^ ± 0.15	7.08^a^ ± 0.32	**
Albumen pH		
9.05^a^ ± 0.05	8.50^b^ ± 0.10	*
Haugh unit		
75.30^b^ ± 0.02	79.70^a^ ± 0.04	**

^a,b^Overall means with different superscripts within a row are significantly different(*P ≤ 0.05, **P ≤ 0.01 and *NS* nonsignificant).

*SPIDES* short period of incubation during egg storage, *S*_*0*_ eggs were stored for 20 days without SPIDES, *S*_*1*_ eggs were stored for 20 days and exposed to heat on the 5th, 10th and 15th day of storage, *DOS* day of storage.

According to Table 1, the extended preincubation period of 20 days and the frequency of SPIDES during this period could not exert a significant influence on parameters such as egg weight, egg shape index, shell weight percentage, and egg surface area, and yolk color at the 20th day of storage (DOS). Conversely, internal characteristics related to egg quality exhibited significant changes due to SPIDES. Specifically, the ratio of albumen to yolk weights, along with albumen and yolk indices, displayed marked enhancements in the S1 group when contrasted with the control group (S0). The S1 group, characterized by reduced albumen weight ratio, albumen index, and Haugh unit, showcased these improvements.

### Egg weight loss%

#### Egg weight loss% during storage

Table 2 displays the findings for egg weight on the 3rd day of storage (3rd-DOS) and illustrates the effect of SPIDES on egg weight loss percentage during the storage period.

**Table 2:** Egg weight at the 3rd DOS and the Effect of SPIDES on egg weight loss % during storage period.

SPIDES (S)		Significance
S_0_	S_1_	
Egg wt at 3rd DOS		
62.15 ± 0.12	64.99 ± 0.37	NS
First wk of storage (3–7)		
0.24^b^ ± 0.04	0.47^a^ ± 0.02	**
Second wk of storage (7–15)		
0.76^b^ ± 0.01	0.89^a^ ± 0.01	**
Third wk of storage		
0.59^b^ ± 0.04	0.90^a^ ± 0.01	**
Total egg weight loss % (3–21)		
1.64^b^ ± 0.08	2.24^a^ ± 0.04	**

^a,b^Overall means with different superscripts within a row are significantly different (*P ≤ 0.05, **P ≤ 0.01 and *NS* nonsignificant).

*SPIDES* short period of incubation during egg storage, *S*_*0*_ eggs were stored for 20 days without SPIDES, *S*_*1*_ eggs were stored for 20 days and exposed to heat on the 5th, 10th and 15th day of storage, *DOS* day of storage.

Table 2 reveals that there was no statistically significant differences in the mean egg weights between the two tested groups (S0 and S1) on the 3rd day of storage. However, it is evident that the eggs' weight loss percentage increased significantly in the SPIDES-treated group, with the S1 group exhibiting the highest total egg weight loss percentage throughout the entire storage period compared to the control group (S0).

#### Egg weight loss during incubation

Table 3 displays the influence of SPIDES and/or TC on the percentage of egg weight loss over the entire incubation duration.

**Table 3:** Effect of Prenatal heating treatments (SPIDES and/or TC) on egg weight loss % during the whole incubation period and egg weight at E18.

SPIDES (S)				Significance		
S_0_	S_1_					
Egg wt loss % 1st wk of incubation						
3.23 ± 0.12	3.22 ± 0.09			NS		
Egg wt loss% 2ndwk of incubation						
2.83 ± 0.10	2.85 ± 0.13			NS		
Egg wt loss% during TC (E14–E18)						
TC_0_	2.04 ± 0.03	2.01 ± 0.03	2.03^B^			
TC_1_	2.48 ± 0.17	2.21 ± 0.26	2.35^A^			
Overall means	2.26	2.11		NS	*	NS
Egg wt at E18						
TC_0_	56.89 ± 0.93	57.90 ± 0.76	57.40^A^			
TC_1_	54.50 ± 0.96	57.53 ± 0.06	57.40^A^			
Overall means	55.69^b^	57.72^a^		NS	*	*

^a,b^Overall means with different superscripts within a row are significantly different (*P ≤ 0.05, **P ≤ 0.01 and *NS* nonsignificant).

*SPIDES* short period of incubation during egg storage, *S*_*0*_ eggs were stored for 20 days without SPIDES, *S*_*1*_ eggs were stored for 20 days and exposed to heat on the 5th, 10th and 15th day of storage, *DOS* day of storage.

The data clearly indicate that SPIDES did not yield a significant effect on egg weight loss percentage during the entire incubation period. Nevertheless, it's noteworthy that the values for egg weight loss percentage exhibited a significant increase during the TC period, specifically from E14 to E18, in the thermal-conditioned groups (TC1S0, TC1S1) when compared to the control groups (TC0S0, TC0S1).

The data presented in Table 3 reveal a direct correlation between egg weight (g) throughout the incubation time and the changes in egg weight loss. Specifically, the results indicate that SPIDES had no significant impact on egg weight (g) at E18. In contrast, TC led to a significant decrease (p < 0.05) in the average egg weight (g) at the end of TC at E18, primarily due to an increase in egg weight loss percentage.

### Productive performance data

Table 4 displays the impact of prenatal heating treatment (SPIDES and TC) on body weight (BW) at hatch and at the marketing age of 35 day of age (DOA).

**Table 4:** Effect of Prenatal heating treatments (SPIDES and/orTC) on live body weight (LBW) at hatch, and 35 DOA .

TC	SPIDES (S)		Overall means	Significance		
	S_0_	S_1_		S	TC	S*TC
Hatch (g)						
TC_0_	40.30 ± 0.17	44.70 ± 0.40	42.50			
TC_1_	39.00 ± 0.29	45.40 ± 0.23	42.20			
Overall means	39.65^b^	45.05^a^		**	NS	*
35 DOA (kg)						
TC_0_	1.54 ± 0.03	2.04 ± 0.02	1.79^B^			
TC_1_	1.90 ± 0.05	2.09 ± 0.03	2.00^A^			
Overall means	1.72^b^	2.05^a^		**	**	*

^a,b^Overall means with different superscripts within a row are significantly different (*P ≤ 0.05, **P ≤ 0.01 and *NS* nonsignificant).

*SPIDES* short period of incubation during egg storage, *S*_*0*_ eggs were stored for 20 days without SPIDES, *S*_*1*_ eggs were stored for 20 days and exposed to heat on the 5th, 10th and 15th day of storage, *35DOA* (day of age) (what means?).

The clear observation is that the prolonged storage duration led to a significant reduction in body weight in the control group in contrast to the SPIDES group.. Nevertheless, no significant differences were observed in body weight at hatch regarding the thermal condition treatment. Furthermore, the average body weight (BW) at 35 days of age (DOA) was significantly increased due to the influence of both SPIDES, TC, and their interaction. SPIDES positively enhanced the productive performance of the broiler chicks. The TC treatment improved the thermal tolerance of the thermally conditioned groups and Led to increased body weights in comparison to the control groups. Remarkably, the synergistic effect of SPIDES and TC had the most prominent impact, as evidenced by the heaviest body weight at hatch (45.40 g) and at 35 days (2.09 kg) achieved for the S1TC1 group compared to 40.3 g and 1.74 kg, respectively, for the control group (S0TC0). Hence, S1TC1 recorded 2.09 kg, S1TC0 recorded 2.04 kg, S0TC1 recorded 1.9 kg, while S0TC0 recorded 1.54 kg (the difference on final weight is very important to mention).

Table 5 displays the impact of prenatal heating interventions (SPIDES and TC) on parameters such as body weight gain (BWG), feed consumption (FC), and feed conversion ratio (FCR) throughout the entire rearing period.

**Table 5:** Effect of Prenatal heating treatments (SPIDES and/or TC) on body weight gain (BWG) , feed consumption (FC), and feed conversion ratio (FCR) of broiler chicks.

TC	SPIDES (S)		Overall means	Significance		
	S_0_	S_1_		S	TC	S*TC
BWG (0–35) DOA (kg)						
TC_0_	1.50 ± 0.03	1.97 ± 0.02	1.74^B^			
TC_1_	1.86 ± 0.05	2.05 ± 0.03	1.96^A^			
Overall means	1.68^c^	2.01^a^		**	**	*
FC (0–35) DOA						
TC_0_	2.42 ± 0.03	3.00 ± 0.06	2.71^B^			
TC_1_	2.64 ± 0.04	3.15 ± 0.04	2.90^A^			
Overall means	2.53^b^	3.08^a^		**	**	**
FCR (0–35) DOA						
TC_0_	1.60 ± 0.01	1.53 ± 0.02	1.57^A^			
TC_1_	1.42 ± 0.02	1.54 ± 0.00	1.48^B^			
Overall means	1.51	1.54		NS	**	**

^a,b^Overall means with different superscripts within a row are significantly different (*P ≤ 0.05, **P ≤ 0.01 and *NS* nonsignificant).

*SPIDES* short period of incubation during egg storage, *S*_*0*_ eggs were stored for 20 days without SPIDES, *S*_*1*_ eggs were stored for 20 days and exposed to heat on the 5th, 10th and 15th day of storage, *DOA* day of age.

The trends in average body weight gain (BWG) results followed the same pattern as described earlier for LBW (as shown in Table 4). This similarity can be attributed to the influence of extended storage duration, as well as the impact of SPIDES and TC during the prenatal stage.

As shown in Table 5, a notably significant reduction in feed consumption (FC) was observed in the untreated control group (S0TC0) in comparison to the other heated treatments (SPIDES and TC). Conversely, the S1TC1 group exhibited the highest feed consumption value over the entire rearing period, suggesting an improved thermotolerance mechanism aimed at achieving enhanced growth performance under such stressful conditions. Regarding the findings related to the feed conversion ratio (FCR), it was observed that the groups subjected to thermal conditioning (TC) exhibited more favorable FCR values compared to the non-TC groups. However, less favorable FCR values were computed for the SPIDES group (S1) in comparison to the S0 group, this could be ascribed to the elevated feed intake observed in the S1 group when contrasted with the S0 group. (You can use the FCR corrected to gain weight, this will show to you the better FCR for group S1TC1 for sure. This group had the highest final weight and gain weight and this influence remarkly the FC and FCR). Done.

### Blood constituents

#### Lipid profile

The lipid profile data collected on the 35th day of age after prolonged exposure to heat stress (as depicted in Table 2) have been presented in Table 6. It is noteworthy to mention that, while the overall plasma cholesterol level did not show a significant impact due to TC, it was significantly elevated in the SPIDES groups compared to the control groups. TC led to a significant reduction in plasma triglyceride levels, whereas the TC groups exhibited higher HDL values compared to the non-thermally influenced groups during conditions of heat stress.

**Table 6:** Effect of Prenatal heating treatments (SPIDES and/or TC) on plasma lipid profile of broiler chicks at 35 DOA.

TC	SPIDES (S)		Overall means	Significance		
	S_0_	S_1_		S	TC	S*TC
Total cholesterol (mg/dl)						
TC_0_	100.00 ± 3.67	108.00 ± 1.64	104.00			
TC_1_	104.00 ± 1.64	104.00 ± 1.64	103.00			
Overall means	102.00^b^	106.00^a^		**	NS	**
Triglycerides (mg/dl)						
TC_0_	46.00 ± 1.63	56.00 ± 1.23	51.00^A^			
TC_1_	38.00 ± 1.22	41.00 ± 1.23	39.50^B^			
Overall means	42.00^b^	48.50^a^		**	**	**
HDL (mg/dl)						
TC_0_	13.00 ± 0.20	16.00 ± 0.16	14.50^B^			
TC_1_	15.00 ± 0.16	17.00 ± 0.12	16.00^A^			
Overall means	14.00^b^	16.5^a^		**	**	**

^a,b^Overall means with different superscripts within a row are significantly different (*P ≤ 0.05, **P ≤ 0.01 and *NS* nonsignificant).

*SPIDES* short period of incubation during egg storage, *S*_*0*_ eggs were stored for 20 days without SPIDES, *S*_*1*_ eggs were stored for 20 days and exposed to heat on the 5th, 10th and 15th day of storage, *DOA* day of age.

#### Blood proteins profile

Plasma levels of total protein, albumin, and globulin on the 35th day of age are displayed in Table 7. Notably, the TC group (TC1) exhibited significantly elevated levels of total protein, albumin, and globulin compared to the control group (TC0). Regarding the impact of SPIDES treatments on plasma protein levels, it was evident that there were varying patterns observed among the different treatment groups. Notably, the S1 group exhibited the highest values for total proteins and globulin levels in comparison to the control group.

**Table 7:** Effect of prenatal heating treatments (SPIDES and/or TC) on plasma protein profile of broiler chicks at 35 DOA.

TC	SPIDES (S)		Overall means	Significance		
	S_0_	S_1_		S	TC	S*TC
Total protein (g/dl)						
TC_0_	5.20 ± 0.04	5.40 ± 0.04	5.25^B^			
TC_1_	5.80 ± 0.04	5.80 ± 1.64	5.90^A^			
Overall means	5.50	5.60		NS	**	**
Albumin (g/dl)						
TC_0_	2.50 ± 0.04	2.40 ± 0.04	2.45^B^			
TC_1_	2.90 ± 0.04	2.60 ± 0.04	2.75^A^			
Overall means	2.70^a^	2.50^b^		NS	**	**
Globulin (g/dl)						
TC_0_	2.70 ± 0.06	3.00 ± 0.06	2.80^B^			
TC_1_	2.85 ± 0.06	3.20 ± 0.06	3.16^A^			
Overall means	2.78^b^	3.10^a^		**	**	**

^a,b^Overall means with different superscripts within a row are significantly different (*P ≤ 0.05, **P ≤ 0.01 and *NS* nonsignificant).

*SPIDES* short period of incubation during egg storage, *S*_*0*_ eggs were stored for 20 days without SPIDES, *S*_*1*_ eggs were stored for 20 days and exposed to heat on the 5th, 10th and 15th day of storage, *DOA* day of age.

#### Aspartate aminotransferase (AST) and alanine aminotransferase (ALT) enzymes

The results displayed in Table 8 depicted how SPIDES and TC influenced the plasma levels of liver function enzymes, specifically aspartate aminotransferase (AST) and alanine aminotransferase (ALT). Surprisingly, in the present study, the liver enzymes, both aspartate aminotransferase (AST) and alanine aminotransferase (ALT), did not show significant alterations due to TC when exposed to heat stress conditions. The findings demonstrated that irrespective of the TC's influence, SPIDES treatment effectively lowered the activity of ALT and AST enzymes. Notably, the S1 group displayed a pronounced decrease, indicating enhanced liver function in comparison to the control group.

**Table 8:** Effect of prenatal heating treatments (SPIDES and/or TC) on plasma enzymatic liver function of broiler chicks at 35 DOA.

TC	SPIDES (S)				Overall means	Significance		
	S_0_	S_1_	S_2_	S_3_		S	TC	S*TC
ALT (U/I)								
TC_0_	5.00 ± 0.41		4.25 ± 0.25		4.63			
TC_1_	5.00 ± 0.41		4.00 ± 0.20		4.50			
Overall means	5.00^a^		4.13^b^			*	NS	NS
AST (U/I)								
TC_0_	253.25 ± 1.89		235.00 ± 16.62		244.13			
TC_1_	272.00 ± 3.24		218.25 ± 12.74		245.13			
Overall means	262.63^a^		225.43^b^			**	NS	**

^a,b^Overall means with different superscripts within a row are significantly different (*P ≤ 0.05, **P ≤ 0.01 and *NS* nonsignificant).

*SPIDES* short period of incubation during egg storage, *S*_*0*_ eggs were stored for 20 days without SPIDES, *S*_*1*_ eggs were stored for 20 days and exposed to heat on the 5th , 10th and 15th day of storage, *DOA* day of age.

### Breast muscles and some internal organ’s relative weights

Table 9 presents the relative weight of breast muscles on the 35th day of age (DOA). It is evident that embryonic thermal conditioning resulted in a significant increase in the relative weight of the entire breast muscles and the percentage of major pectoralis muscles. However, SPIDES did not have any noticeable impact. Furthermore, the relative weight of minor pectoralis muscles remained unchanged due to both TC and SPIDES.

**Table 9:** Effect of Prenatal heating treatments (SPIDES and/or TC) on breast muscles relative weights at 35 DOA.

TC	SPIDES (S)		Overall means	Significance		
	S_0_	S_1_		S	TC	S*TC
Whole breast muscles						
TC_0_	22.32 ± 0.79	24.66 ± 1.22	23.48^B^			
TC_1_	26.52 ± 0.19	25.54 ± 0.32	26.03^A^			
Overall means	24.42	25.16		NS	*	*
Major pectoralis %						
TC_0_	18.64 ± 0.73	20.48 ± 0.93	19.55^B^			
TC_1_	22.19 ± 0.21	21.68 ± 0.34	21.93^A^			
Overall means	20.25	21.16		NS	**	*
Minor pectoralis %						
TC_0_	3.68 ± 0.12	4.18 ± 0.31	3.94			
TC_1_	4.33 ± 0.37	3.86 ± 0.17	4.12			
Overall means	4.01	4.02		NS	NS	NS

^a,b^Overall means with different superscripts within a row are significantly different (*P ≤ 0.05, **P ≤ 0.01 and *NS* nonsignificant).

*SPIDES* short period of incubation during egg storage, *S*_*0*_ eggs were stored for 20 days without SPIDES, *S*_*1*_ eggs were stored for 20 days and exposed to heat on the 5th, 10th and 15th day of storage, *DOA* day of age.

### Metabolic hormone plasma level

The data displayed in Table 10 depicts the plasma T3 levels influenced by SPIDES, both with and without TC, at both hatch and the marketing age (35 DOA). The findings indicated a significant (P ≤ 0.05) reduction in plasma T3 levels at the hatch for the TC groups in comparison to those without TC. The application of SPIDES treatment led to a significant reduction (P ≤ 0.05) in plasma T3 levels at hatching when compared to the remaining groups.. Plasma T3 levels were assessed at the marketing age of 35 days post-hatch, during conditions of chronic heat stress. The results revealed a significant reduction in T3 levels among the TC groups when compared to the control group.

**Table 10:** Effect of Prenatal heating treatments (SPIDES and/or TC) on T_3_ hormones plasma level at hatch and 35 DOA.

TC	SPIDES (S)		Overall means	Significance		
	S_0_	S_1_		S	TC	S*TC
Hatch						
TC_0_	1.56 ± 0.10	1.24 ± 0.03	1.39^B^			
TC_1_	1.67 ± 0.12	1.26 ± 0.02	1.45^A^			
Overall means	1.62^a^	1.25^b^		**	**	**
35 DOA						
TC_0_	1.34 ± 0.02	1.42 ± 0.02	1.36^A^			
TC_1_	1.30 ± 0.01	1.33 ± 0.02	1.31^B^			
Overall means	1.32^b^	1.38^a^		**	**	**

^a,b^Overall means with different superscripts within a row are significantly different (*P ≤ 0.05, **P ≤ 0.01 and *NS* nonsignificant).

*SPIDES* short period of incubation during egg storage, *S*_*0*_ eggs were stored for 20 days without SPIDES, *S*_*1*_ eggs were stored for 20 days and exposed to heat on the 5th, 10th and 15th day of storage, *DOA* day of age.

### Histological and cytological study

#### Histological and cytological observations of breast muscle

**Figure 1:**
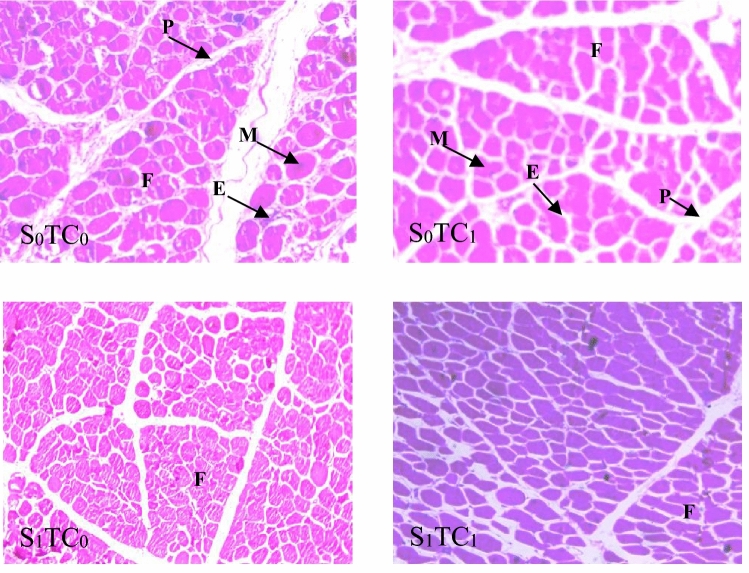
Transverse section through pectoralis breast muscle from birds of different treatments at 35 DOA. *F* fascicles, *E* endomysium, *M* myocytes, *P* perimysium (H & E ×100). S_0_TC_0_ = control no SPIDES & incubation under normal condition, *S*_*0*_*TC*_*1*_ control no SPIDES & prenatal thermal conditioning, *S*_*1*_*TC*_*0*_ SPIDES & incubation under normal condition, *S*_*1*_*TC*_*1*_ SPIDES & prenatal thermal conditioning, *TC* prenatal thermal conditioning, *SPIDES* short period of incubation during egg storage period.

Figure 1 illustrates the microscopic arrangement of major pectoralis muscles across distinct treatment groups. Through histological examination, it becomes apparent that sections obtained from various treatments exhibit noteworthy distinctions. As a result, the examination and measurement of cytological variances emerge as a superior method for distinguishing between different experimental groups, given their display of characteristic skeletal muscular structures.

The count of myocytes per field witnessed a significant increase due to both SPIDES and embryonic thermal conditioning. This rise in myocyte count signifies enhanced lateral growth performance. Furthermore, during the storage period, SPIDES exhibited a positive impact on augmenting the number of myocytes per field.

Regarding the dimensions of myocytes (both in terms of large and small diameters), their thickness (*D*$\overline{x}$), and cross-sectional area (*SA*), the present findings indicate that the elevated myocyte count observed in the thermally conditioned and SPIDES-exposed groups is correlated with reductions in myocyte dimensions, mean thickness, and cross-sectional area. These reductions were found to be statistically significant when compared to the control groups (Table 11).

**Table 11:** Effect of prenatal heating treatments (SPIDES and/or TC) on breast muscles histological and cytological traits at 35 DOA.

TC	SPIDES (S)		Overall means	Significance		
	S_0_	S_1_		S	TC	S*TC
Myocytes numbers/field						
TC_0_	81.92 ± 7.21	168.50 ± 8.01	125.11^B^			
TC_1_	127.27 ± 5.30	161.67 ± 5.95	144.47^A^			
Overall means	102.71^b^	163.77^a^		**	**	**
Large diameter ($LD$ µm)						
TC_0_	50.47 ± 1.01	51.58 ± 0.87	51.03^A^			
TC_1_	42.81 ± 0.69	43.40 ± 0.49	43.11^B^			
Overall means	46.41	46.58		NS	**	**
Small diameter ($SD$ µm)						
TC_0_	34.14 ± 0.62	25.95 ± 0.47	30.05			
TC_1_	34.63 ± 0.51	31.85 ± 0.34	33.24			
Overall means	34.40^a^	29.56^b^		**	NS	**
Mean thickness ($D\overline{x}$ µm)						
TC_0_	42.30 ± 0.65	38.76 ± 0.52	40.52^A^			
TC_1_	38.72 ± 0.52	37.63 ± 0.33	38.18^B^			
Overall means	40.40^a^	38.07^b^		**	**	**
Shape index ($SI$) ($LD$/$SD$ ratio)						
TC_0_	1.51 ± 0.04	2.05 ± 0.05	1.79^A^			
TC_1_	1.25 ± 0.02	1.38 ± 0.02	1.32^B^			
Overall means	1.37^b^	1.64^a^		**	**	**
Cross section area ($SA$ µm^2^)						
TC_0_	1364.32 ± 42.41	1055.52 ± 29.31	1209.82^A^			
TC_1_	1178.43 ± 31.64	1091.27 ± 19.48	1134.75^B^			
Overall means	1265.80^a^	1077.38^b^		**	**	**

^a,b^Overall means with different superscripts within a row are significantly different (*P ≤ 0.05, **P ≤ 0.01 and *NS* nonsignificant).

*SPIDES* short period of incubation during egg storage, *S*_*0*_ eggs were stored for 20 days without SPIDES, *S*_*1*_ eggs were stored for 20 days and exposed to heat on the 5th, 10th and 15th day of storage, *DOA* day of age.

## Discussion

Extended storage periods of eggs, even when kept in ideal storage conditions, can significantly impair egg quality. This deterioration is evident in the subsequent quality and performance of the chicks that hatch from these eggs^4^. This effect might arise from the potential loss of albumen solvent from the albumen to the yolk due to the degradation of the ovomucin-lysozyme complex after prolonged storage periods, as observed by^27^. This breakdown of the ovomucin-lysozyme complex is manifested in the reduction of albumen height, which, in turn, influences the Haugh unit, leading to a significant decrease during extended storage periods. Furthermore^28^, conducted a study revealing that subjecting eggs to SPIDES at 37.5 °C for 4 h during an extended storage period of 10 days resulted in significantly elevated percentages of albumen weight and Haugh Unit values in comparison to the control group. In contrast, the research conducted by^7^ found that implementing SPIDES for a duration of 2 h at 36 °C, until the eggshell temperature (EST) reached 32–34 ℃ during extended storage periods ranging from 6 to over 21 days of refrigerated storage, did not yield any discernible impact on internal egg quality. In relation to yolk color, Our results are consistent with those reported in the study by^27^, who indicated that yolk color remained unaffected by prolonged storage. They attributed any potential alteration in yolk color to the antioxidant content in the hens' diet and other environmental factors.

It has been hypothesized that^27^ the significant increase in yolk ratio, along with the concurrent decline in yolk index value—a trend consistent with our current results, particularly in the control group relative to the SPIDES group (S1)—may be ascribed to the transfer of water from the albumen to the yolk, which could be linked to a weakening of the vitelline membrane strength (VMS). This weaker membrane could render the yolk more vulnerable to breakage, facilitating the gradual infiltration of water from the albumen. Consequently, the yolk percentage increased compared to the albumen, resulting in the characteristic mottled flattened appearance of the yolk, as manifested in the reduced yolk index of the control group compared to the S1 group.

Conversely, the decline in albumen pH might be due to the evaporation and carbon dioxide exchange from the eggs, shifting the bicarbonate buffer system towards CO_2_ production, consequently raising the albumen pH^6^.

The ratio of weight loss in chicken eggs exhibited a noteworthy increase due to extended storage, as evidenced by the studies conducted by^29^. Furthermore, as indicated by^30^, the egg weight loss experienced a substantial rise with longer storage periods, with an estimated increase exceeding 10% for eggs stored for 35 days. This escalating trend in egg weight loss percentages over extended storage durations could be attributed to the evaporation-driven loss of solvents from the egg contents through the eggshell^27^. Concerning with SPIDES effect,^22^ reported that subjecting the Cobb 500 broiler breeder flock to preheating for durations of 6 or 12 h during extended storage periods of 9 to 12 days led to a significant increase in egg weight loss percentages in comparison to non-heated groups (control). In opposition to the outcomes observed in the current investigation^31^ reported that pre-incubation treatments applied to 37-week-old broiler breeder eggs had no significant impact on egg weight losses throughout the entire storage period.

Observations made by^32^ unveiled that the prolonged storage of Guinea fowl eggs for a period of 10 days led to a significant rise in egg weight loss during storage, in contrast to eggs stored for just 4 days. This underscores a direct correlation between the duration of egg storage and the rate of egg weight loss.

our findings align with the results of^33^, As indicated by their research, which involved raising the eggshell temperature to 38.9 °C for the Cobb 500 commercial breeder flocks aged 37 to 45 weeks, starting from E 8 onwards, led to significant percentages of egg weight loss at E18. However, this effect did not extend to create substantial differences in the overall egg weight. The current observation of non-significance in egg weight loss percentage during incubation due to SPIDES corresponds with the conclusions drawn by^34,35^, who determined that a prolonged storage period for chicken eggs did not have a noteworthy impact on the percentages of egg weight loss during the incubation process. Moreover, when examining the incubation-related egg weight loss of Cobb 500 broiler breeder flocks that were preheated for 6 or 12 h after being stored for 9 to 12 days, there was no statistically significant distinction from the non-heated (control) groups, as indicated by^22^.

It is evident that an extended storage duration resulted in a decrease in the body weight of the control group compared to the SPIDES groups. In this context,^33^ observed that elevating the eggshell temperature to 38.9 °C for 6 h during the incubation of Cobb 500 commercial breeder flocks aged 37 to 45 weeks, starting from E8 onwards, led to extended chick length after hatching but reduced body weight compared to the control group. In contrast to our findings,^36^ demonstrated a significant improvement in chick weight at hatch following thermal conditioning at 40 °C for 4 h per day from E14 to E17, in comparison to the control group.

Furthermore, average body weights (BW) experienced a significant increase at 35 days of age (DOA) due to the effect of both SPIDES and thermal conditioning (TC), as well as their interaction. SPIDES positively impacted the productive performance of broiler chicks, with its effectiveness being linked to the timing of SPIDES. The TC treatment displayed the capability to enhance the heat tolerance of the thermally conditioned groups, leading to higher body weights when contrasted with the control groups. Notably, the combined effect of SPIDES and TC was even more pronounced, evident in the heaviest body weight at hatch (45.40 g) achieved by the S1TC2 group when contrasted with the control group(S0TC0) with a weight of 40.3 g. In alignment with the present results, El-Garhy^37^ identified a significant augmentation in live body weight (LBW) and the rate of body weight gain (BWG) both at hatch and at the 5th week of age when compared with untreated groups. Partially concurring with the current findings, Tona et al.^38^ found that extended storage of eggs from young breeders (35 weeks of age) significantly decreased the relative weight gain of 7-day-old chicks.

Regarding the feed conversion ratio (FCR) results, it was observed that the groups subjected to thermal conditioning (TC) demonstrated better FCR than the non-TC groups. However, the SPIDES group (S1) exhibited poorer FCR compared to the S0 group. This observation was attributable to the higher feed consumption (FC) observed in the S1 treatment group in contrast to the S0 group. (review this affirmation with correted FCR). Done.

In accordance with our findings, previous research conducted by^39^ revealed that chicks originating from thermally conditioned eggs displayed significantly higher live body weight (LBW), body weight gain (BWG), and improved feed conversion ratio (FCR) both at hatch and throughout subsequent ages, in comparison with the control group. Similarly, Meteyake et al.^40^ showed that exposing Ross eggs to thermal conditioning at 39.5 °C for 12 h daily, starting from embryonic day 7 (E7) and continuing until embryonic day 16 (E16), led to improved final body weight, reduced feed conversion, and lowered feed intake.

In contrast, studies by^41–43^, demonstrated that a decrease in body weight from hatching until 6 weeks of age for chicks hatched from eggs exposed to different thermal conditioning regimes. Sengor et al.^41^ exposed eggs to TC at 39 °C for 2 h per day at E14 and E15, while^42^ subjected eggs to 39.6 °C or 40.6 °C. Similarly, Zaboli et al.^43^ found that thermal conditioning of Ross 308 eggs at 39.5°C and 65% relative humidity for 12 h per day from E7 to E16 led to a decrease in body weight when contrasted with the control group.

Contrastingly, In the later phases of the incubation process, studies by^44,45^ had no significant variances in body weight, feed consumption, and feed conversion ratio between control chicks and those subjected to embryonic thermal conditioning. Furthermore, neither exposing Hubbard eggs to 38.8°C for 6 or 18 h per day from E10 to E18^46^ nor subjecting them to 39°C and 65% relative humidity for 18 h per day from E0 to E18^47^ had a significant impact on body weight at hatch and up to 4 weeks of age.

It was proposed that stress might lead to an elevation in overall cholesterol levels by boosting LDL while reducing HDL, which promotes the onset of cardiovascular disorders^48^. The same finding was obtained under conditions of prolonged exposure to heat stress at 34 °C for 8 h daily from the 22nd to the 42nd h^49^, as well as in instances where a broiler was subjected to extended periods of heat stress at 36 °C^50^. Similarly, under conditions of cyclic stress, there was a significant rise in plasma cholesterol levels^51^.

Triglyceride plasma levels were notably lowered by TC, in contrast to the higher HDL values observed in TC groups compared to non-thermally influenced groups under heat stress conditions. This indicates that the TC groups adapted metabolically to the heat stress^52^. Furthermore, they noted a significant elevation in triglyceride levels, total cholesterol levels, and low-density lipoprotein cholesterol levels in broiler chicks at 42 days old as a result of prolonged exposure to chronic heat stress at 32 °C from the 22nd to the 42nd day of age. On the other hand, cyclic heat stress at 36 °C from the 16th to the 42nd day of age had no significant effect on plasma triglycerides, HDL, or LDL levels^51^.

The plasma levels of lipid parameters due to the influence of SPIDES displayed inconsistent value trends, suggesting that the variations in lipid parameters were primarily influenced by chronic heat stress, as well as the interaction between SPIDES and TC. The S1TC1 group exhibited significantly higher plasma HDL levels and lower TG levels when contrasted with the control group S0TC0, indicating the enhancing effect of SPIDES on plasma lipid profile and lipid metabolism under heat stress conditions. These findings align with those of^32^ who found that storing Guinea fowl eggs for 15 days increased total cholesterol and triglyceride concentrations compared to chicks hatched from eggs stored for three days.

The significant elevated levels of total protein and albumin observed in the (S1TC1) group, as opposed to the control (S0TC0) group, potentially indicate the heightened metabolic protein activity within the S1TC1 group due to its increased feed consumption (FC) under heat stress circumstances. This observation underscores the synergistic collaboration between TC and SPIDES in enhancing liver protein synthesis, metabolic efficiency, and overall chick performance when faced with heat stress conditions^36,53^.

In the present investigation, it was unexpectedly observed that the levels of both liver enzymes remained largely unchanged by TC when subjected to conditions of heat stress. This lack of statistically significant alteration might be attributed to the interplay between the varied performance of chicks hatched from SPIDES groups and their adaptive response to heat stress, wherein they seem to have developed a viable metabolic mechanism to enhance their resilience against heat-induced challenges. Notably, it is worth highlighting that irrespective of TC's impact, the administration of SPIDES treatment was able to considerably diminish the activity of ALT and AST enzymes. This effect was particularly pronounced in the S1 group, indicating improved liver functionality when contrasted with the control group. In this context, it is worth noting that^54^ documented that subjecting broiler chicks to thermal stress at 34 °C for a duration of 8 h resulted in heightened plasma levels of AST and ALT. Furthermore,^55^ noted that elevated ambient temperatures had the potential to trigger liver impairment, leading to an escalation in serum AST and ALT concentrations.

Our results are consistent with the discoveries made in the study by^56^, who illustrated that subjecting embryos to temperature conditioning (TC) at 39.5 °C for either 3 or 6 h per day during the later stages of incubation (E16 to E18) resulted in enhanced development of the major pectoralis muscle in chicks by the 35th day of age, when contrasted with the control group. Additionally, applying TC at 39.5 °C for 12 h per day, spanning from E7 to E16, significantly improved the yield of breast muscle and concurrently reduced the relative percentage of abdominal fat at the age of market readiness. This was corroborated by the studies of^57–59^.

In a similar vein, a study by^39^ unveiled that subjecting broiler eggs to temperature conditioning at 38.6 °C and 65% relative humidity for 6 h per day, starting from E16 to E8, resulted in a rise in the weight of breast muscles upon reaching the market age.

The thyroid hormone holds a pivotal function in avian thermogenesis, facilitating thermoregulation by regulating the generation of metabolic heat. This regulation is essential for sustaining a consistent body temperature across varying thermal environments, as emphasized by^60^. The results indicated a significant (P ≤ 0.05) reduction in plasma T3 levels at hatch for the TC groups in comparison to those without TC.

This reduction in T3 levels in the TC chicks corresponded with a lowered metabolic rate, leading to decreased heat production in response to the heat stress conditions. The findings indicated a significant (P ≤ 0.05) reduction in plasma T3 levels at hatch within the TC groups compared to the non-TC groups. This decrease suggests a higher metabolic rate in TC1 chicks in contrast to the control group (TC0), which in turn promoted improved growth performance. This observation has been attributed to embryonic Thermal manipulation between developmental days E6 to E16, influencing the establishment of the brain's thermoregulatory center. This alteration of the 'setpoint' for thermotolerance systems subsequently influences the metabolic rate under similar environmental conditions, as elucidated by^60^. Energy usage, both in a general sense and specifically within domestic fowl, can be categorized into two main aspects: maintenance and production. In warm-blooded organisms (homeotherms), maintaining a stable body temperature (Tb) requires a significant portion of energy for basic upkeep. Consequently, if we can reduce the energy needed for maintenance while maintaining a consistent overall energy consumption, it's probable that more energy would be accessible for productive purposes. Alternatively, another approach could involve a combination of lowering the energy required for maintenance along with an overall reduction in energy consumption^61^. Numerous researchers have employed embryonic thermal manipulation at around 39.5 °C for 6 to 12 h per day to enhance thermotolerance in the face of heat stress. This manipulation has been associated with changes in circulating T3 and T4 plasma levels, indicating an enhancement in thermotolerance under thermal stress conditions^46,62,63^.

Conversely, applying the SPIDES treatment during the 20 days of incubation showed a significant reduction (P ≤ 0.05) in T3 plasma levels at hatch compared to the other groups. This effect might be attributed to the earlier hatching time of the S1 group. The accelerated embryonic metabolic rate before hatch could be responsible for aiding the embryo in transitioning to lung ventilation and engaging its respiratory and leg muscles to hatch. Given that the S1 group hatched earlier, their T3 plasma levels likely returned to normal more swiftly compared to the chicks that hatched later.

Assessment of estimated plasma T3 levels at the marketing age of 35 days, under chronic heat stress conditions, revealed a significant reduction in TC groups when contrasted with the control group. This reduction in T3 levels among TC chicks indicates a lowered metabolic rate and subsequently reduced heat production under heat stress. This aligns with the observation that broiler chickens tend to decrease both the size and activity of their thyroid gland under high ambient temperatures^64^. Additionally, conducting chronic incubation at a temperature of 39.0 °C for 3 h from embryonic days E6 to E8 Led to a reduction in the plasma levels of T3^57,65^ noted a significant reduction in both T3 levels and the T3/T4 ratio, alongside an elevation in plasma T4 levels, as an outcome of subjecting embryos to temperature conditioning (TC) at 39.5 °C for 12 h daily, encompassing the period from E7 to E16. Similarly, Al-Zghoul et al.^46^ noted that elevating the surrounding temperature to 41.0 °C for 6 h per day at 3, 7, or 42 days of age led to a significant reduction in plasma T3 levels at 42 days of age in contrast to normal incubation or control conditions.

However, it should be noted that embryonic thermal manipulation at 39 °C for 3 h per day from E16 to E18 of embryonic development increased the concentration of free T3 hormone on E19 and decreased the concentration of free T4 hormone in the embryo's blood, as opposed to the control group^66^. In contrast, embryonic TC at 40 °C for 4 h per day from E14 to E17 had no significant effect on T3 serum levels in Golden Montazah chickens^36^.

The count of myocytes per field witnessed a significant rise due to both SPIDES and embryonic thermal conditioning. This surge in myocyte numbers signifies enhanced lateral growth performance. Additionally, during the storage period, SPIDES exhibited a beneficial impact on augmenting myocyte count per field.

In terms of myocyte dimensions, encompassing both large and small diameters, thickness, and cross-sectional area, the present findings suggest that the escalated count of myocytes within thermally conditioned and SPIDES-exposed groups is mirrored in their myocyte dimensions, mean thickness, and cross-sectional area. Notably, these measurements were significantly lesser when compared to the control groups.

It is widely recognized that muscle fiber count, dimensions, and fiber-type distribution are intricately interlinked^67^. Muscle function in mature animals is significantly influenced by factors such as type and number of muscle fiber, which subsequently impact fiber size. The total muscle mass is determined by the aggregate of the myofiber count, their cross-sectional area, and their length^68^.

Through histological examination and cytological measurements of pectoralis muscles in this current investigation, it is implied that subjecting broiler eggs to temperature conditioning throughout the latter stages of embryonic development leads to an increase in the count of myocytes within the pectoralis muscles upon reaching the marketing age.

In previous investigations, it was reported that subjecting embryos to thermal conditioning (TC) between developmental stages E16 and E18 led to the stimulation of myonuclei proliferation. As a result, the number of myonuclei in the breast muscles of newly hatched broiler chicks increased. This phenomenon of myonuclei hyperplasia subsequently brought about an enlargement of the pectoralis muscles by augmenting the count of muscle fibers, or myocytes. This, in turn, contributed to an increased yield of breast muscles at the time of market age. This concept aligns with the outcomes of^69^.

Similarly,^70^ demonstrated that deviating from the standard incubation temperature range could directly impact the molecular processes essential for myoblast proliferation and differentiation. Alternatively, it might indirectly influence myogenesis by promoting metabolic activity and encouraging embryonic movement within the egg. In a similar vein, exposing late-term embryos to elevated temperatures (38.5 °C or 39.5 °C for 3 or 6 h per day) was found to enhance both myofiber diameter and myoblast proliferation, thereby promoting overall muscle growth at the market stage, as documented by^56,71^.

Additionally, these findings align with^69^, who demonstrated that subjecting embryos to heat conditioning at 39.5 °C for 5 h per day during the E16 to E18 timeframe significantly amplified the count of myocytes. This increase was observed to influence myocyte dimensions and cross-sectional area, leading to substantial post-hatch growth among the thermally conditioned groups compared to the control groups.

The improved histological structure of the thermally conditioned and S1 groups is further substantiated by the higher relative weights of breast muscles observed at market age in the thermally conditioned and S1 groups, particularly in the S1TC1 treatment when compared to the other groups.

## Conclusion

SPIDES treatment group displayed marked enhancements in internal characteristics related to egg quality, SPIDES positively enhanced the productive performance of the broiler chicks. Moreover, The TC treatment improved the thermal tolerance of the thermally conditioned groups and led to increased body weights. Remarkably, the synergistic effect of SPIDES and TC had the most prominent impact. The count of myocytes had a significant increase due to both SPIDES and embryonic thermal conditioning, it could be concluded that SPIDES and TC have beneficial effects on pre- and post-hatch characteristics of broiler chicks up until the marketing age. Additionally, TC techniques improve chick performance, particularly under conditions of heat stress, and enhance the yield of breast meat in later stages of life.

## Data Availability

The datasets employed and/or examined in the present study can be obtained from the corresponding author upon a reasonable request.

## Abbreviations

AST: Aspartate aminotransferase
ALT: Alanine aminotransferase
BW: Body weights
BWG: Body weight gain
FC: Feed consumption
FCR: Feed conversion ratio
SPIDES: Short periods of incubation during egg storage
TC: Thermal conditioning
T3: Triiodothyronine
TG: Triglyceride
HDL: High density lipoprotein
